# The cardiovascular risk profile of middle age women previously diagnosed with premature ovarian insufficiency: A case-control study

**DOI:** 10.1371/journal.pone.0229576

**Published:** 2020-03-05

**Authors:** Marlise N. Gunning, Cindy Meun, Bas B. van Rijn, Nadine M. P. Daan, Jeanine E. Roeters van Lennep, Yolande Appelman, Eric Boersma, Leonard Hofstra, Clemens G. K. M. Fauser, Oscar L. Rueda-Ochoa, Mohammad A. Ikram, Maryam Kavousi, Cornelis B. Lambalk, Marinus J. C. Eijkemans, Joop S. E. Laven, Bart C. J. M. Fauser, on behalf of the CREW consortium

**Affiliations:** 1 Department of Reproductive Medicine and Gynecology, University Medical Center Utrecht, University of Utrecht, Utrecht, Utrecht, the Netherlands; 2 Division of Reproductive Endocrinology and Infertility, Department of Obstetrics and Gynecology, Erasmus University Medical Center, Rotterdam, South Holland, the Netherlands; 3 Department of Obstetrics, Wilhelmina Children’s Hospital Birth Center, University Medical Center Utrecht, Utrecht, Utrecht, the Netherlands; 4 Division Obstetrics and Fetal Medicine, Department of Obstetrics and Gynecology, Erasmus University Medical Centre, Rotterdam, South Holland, the Netherlands; 5 Department of Internal Medicine, Erasmus Medical Center, Rotterdam, South Holland, the Netherlands; 6 Department of Cardiology, Amsterdam UMC, VU University Amsterdam, Amsterdam, North Holland, the Netherlands; 7 Cardiovascular Research School COEUR, Rotterdam, South Holland, the Netherlands; 8 Department of Epidemiology, Erasmus Medical Center, Rotterdam, South Holland, the Netherlands; 9 Department of Cardiology, Erasmus Medical Center, Rotterdam, South Holland, the Netherlands; 10 Cardiology Center Netherlands, Utrecht, Utrecht, the Netherlands; 11 School of Medicine, Universidad Industrial de Santander, Bucaramanga, Santander, Colombia; 12 Department of Neurology, Erasmus Medical Center, Rotterdam, South Holland, The Netherlands; 13 Department of Obstetrics and Gynecology, Amsterdam University Medical Center – location VUmc, Amsterdam, North Holland, the Netherlands; 14 Julius Centre for Health Sciences and Primary care, University Medical Center Utrecht, Utrecht, Utrecht, the Netherlands; Tongji Med College, HUST, CHINA

## Abstract

**Background:**

Cardiovascular disease (CVD) is the leading cause of death in women worldwide. The cardiovascular risk profile deteriorates after women enter menopause. By definition, women diagnosed with premature ovarian insufficiency (POI) experience menopause before 40 years of age, which may render these women even more susceptible to develop CVD later in life. However, prospective long-term follow up data of well phenotyped women with POI are scarce. In the current study we compare the CVD profile and risk of middle aged women previously diagnosed with POI, to a population based reference group matched for age and BMI.

**Methods and findings:**

We compared 123 women (age 49.0 (± 4.3) years) and diagnosed with POI 8.1 (IQR: 6.8–9.6) years earlier, with 123 population controls (age 49.4 (± 3.9) years). All women underwent an extensive standardized cardiovascular screening. We assessed CVD risk factors including waist circumference, BMI, blood pressure, lipid profile, pulse wave velocity (PWV), and the prevalence of diabetes mellitus, metabolic syndrome (MetS) and carotid intima media thickness (cIMT), in both women with POI and controls. We calculated the 10-year CVD Framingham Risk Score (FRS) and the American Heart Association’s suggested cardiovascular health score (CHS). Waist circumference (90.0 (IQR: 83.0–98.0) versus 80.7 (IQR: 75.1–86.8), p < 0.01), waist-to-hip ratio (0.90 (IQR: 0.85–0.93) versus 0.79 (IQR: 0.75–0.83), p < 0.01), systolic blood pressure (124 (IQR 112–135) versus 120 (IQR109-131), p < 0.04) and diastolic blood pressure (81 (IQR: 76–89) versus 78 (IQR: 71–86), p < 0.01), prevalence of hypertension (45 (37%) versus 21 (17%), p < 0.01) and MetS (19 (16%) versus 4 (3%), p < 0.01) were all significantly increased in women with POI compared to healthy controls. Other risk factors, however, such as lipids, glucose levels and prevalence of diabetes were similar comparing women with POI versus controls. The arterial stiffness assessed by PWV was also similar in both populations (8.1 (IQR: 7.1–9.4) versus 7.9 (IQR: 7.1–8.4), p = 0.21). In addition, cIMT was lower in women with POI compared to controls (550 μm (500–615) versus 684 μm (618–737), p < 0.01). The calculated 10-year CVD risk was 5.9% (IQR: 3.7–10.6) versus 6.0% (IQR: 3.9–9.0) (p = 0.31) and current CHS was 6.1 (1.9) versus 6.5 (1.6) (p = 0.07), respectively in POI versus controls.

**Conclusions:**

Middle age women with POI presented with more unfavorable cardiovascular risk factors (increased waist circumference and a higher prevalence of hypertension and MetS) compared to age and BMI matched population controls. In contrast, the current study reveals a lower cIMT and similar 10-year cardiovascular disease risk and cardiovascular health score. In summary, neither signs of premature atherosclerosis nor a worse cardiovascular disease risk or health score were observed among middle age women with POI compared to population controls. Longer-term follow-up studies of women of more advanced age are warranted to establish whether women with POI are truly at increased risk of developing CVD events later in life.

**Trial registration:**

ClinicalTrials.gov Identifier: NCT02616510.

## Introduction

Cardiovascular disease (CVD) continues to be the leading cause of morbidity and mortality in women, affecting up to 36% of all women worldwide [1,2]. A distinct increase in CVD risk is commonly observed from menopause onwards [3,4]. Aggregated data concerning women aged 40 years or older provide evidence that an early age of menopause is associated with a higher mortality risk due to CVD (follow-up ranging from 10–24 years) [5–10]. Several large sample size epidemiological studies proclaim a reduced life expectancy in women entering menopause before 40 years of age [8,11,12].

The most convincing body of evidence concerning an association between early menopause and hard CVD endpoints is based on observations in women undergoing bilateral oophorectomy [13–15]. Women exposed to a prophylactic bilateral oophorectomy for the prevention of ovarian cancer before age 45 years, exhibit a distinctly increased risk for CVD, CVD-mortality and all-cause mortality, compared to age-matched controls. A sub-group of women who did not use estrogen replacement therapy after surgery was most affected [13–15]. These observations await confirmation in women with a confirmed POI diagnosis due to non-surgical reasons. Prospective long-term follow up data of well phenotyped women with POI are scarce.

Women with POI suffer from the most extreme form of early menopause, by definition before 40 years of age. This condition is observed in 1–2% of all women worldwide [16]. POI may be caused by chromosomal defects, genetic disorders, autoimmune diseases, infectious diseases or iatrogenic conditions [17]. However, in the vast majority of women with POI the underlying cause remains unknown [18].

The hallmark of POI is a prolonged state of estrogen deficiency affecting women in many different ways, such as psycho-social and sexual wellbeing, infertility and decreased bone mineral density [19,20]. An earlier cross sectional study related the decline of estradiol levels across consecutive stages of the menopausal transition in 132 healthy women aged 22–70 years, to increased vascular aging. The flow mediated dilation (FMD) was used as a proxy measure for vascular aging [21]. More atherogenic lipid profiles were also reported in women with POI [22]. In addition, POI may also act as an independent risk factor for ischemic heart disease and overall CVD, in line with the previously reported negative association between age of menopause and CVD risk [23,24]. The aggregated impact of these components on CVD risk remains elusive and evidence in the younger age groups is still scarce.

The current analysis represents a distinct extension of our initial preliminary observations in women with POI published earlier [25]. In the previous study we compared the two extreme phenotypes with each other: women with POI versus women of approximately similar age with premenopausal status [25]. We observed increased cardiovascular risk factors in women with POI compared to premenopausal women of comparable age, but no sign of increased subclinical atherosclerosis. So far, we did not perform any risk prediction for future CVD in women with POI at middle age. We also did not provide evidence on which health metrics are most eligible to better, for improvement of cardiovascular health in our study population. The novelty of the current study lies in: I) the selected control group, II) the investigation of vascular stiffness, and III) the risk and health scores calculated in this study population. The aim of the current study is to compare a larger group of Dutch middle age women previously diagnosed with POI with, in the current study, an individually age- and BMI-matched Dutch population based reference cohort at, regardless of their menopausal status. Outcomes of interest were: subclinical atherosclerosis, 10-year cardiovascular risk score (Framingham Risk Score (FRS) and a cardiovascular health score [26,27].

## Materials and methods

### Patients

Women diagnosed with POI were selected from the large Dutch multi-center study involving patients presenting with oligomenorrhea/amenorrhea. According to protocol, all these women underwent a detailed and standardized history taking, physical examination and endocrine assessment (so called COLA screening; registration number NCT02309047), as previously described in detail elsewhere [28]. POI was defined as the occurrence of secondary amenorrhea ≥ 4 months before the age of 40 years, along with FSH levels above 40 IU/L [18]. At the time of establishing POI diagnosis by a gynecologist with the fore mentioned criteria, patients were aged < 40 years. At the time of confirmation by the COLA screening patients were aged 39 years (IQR: 37–43) and last menstruation was experienced at an average age of 35 years (IQR: 32–38).

For the current study, we included women ≥ 40 years of age and previously diagnosed with spontaneous (non-surgical) POI. These women were recruited from three Dutch university medical centers: University Medical Center Utrecht, Erasmus Medical Center Rotterdam and Amsterdam University Medical Center (location VUMC). This study was approved by the local ethics review board of the University Medical Center Utrecht and registered at www.clinicaltrials.gov (NCT02616510). Written informed consent was obtained from all participants.

### Population based controls

We selected age- and BMI-matched controls from the population-based cohort Rotterdam Study, initiated in 1990. The Rotterdam Study aims to investigate the incidence and risk factors for numerous chronic diseases conditions, including CVD in elderly [29]. We used data of the third cohort of the Rotterdam Study (RS-III), in which inhabitants of the municipality of Ommoord, Rotterdam aged > 45 years, were included between 2006 and 2008, as published previously. The rationale and design of this study have been described in detail elsewhere [30]. Controls were included regardless of their menopausal state, since we did not aim to compare two extreme phenotypes of the menopausal spectrum, as in our previous study [25]. All participants provided written informed consent to participate and to obtain information from their treating physicians. This population based cohort study has been approved by the medical ethics committee according to the Population Screening Act: Rotterdam Study, executed by the Ministry of Health, Welfare and Sports of the Netherlands (registration number: NL6645).

### Endocrine and cardiovascular assessment of women with POI and controls

Cardiovascular screening by cardiologists or cardiovascular oriented internal medicine doctors was performed at three clinics: Erasmus Medical Center Rotterdam, Bergman Clinics Bilthoven and Cardiology Center Utrecht. Detailed information regarding the screening procedure has been previously described [25]. In brief, we assessed general medical, obstetric and family history, educational level, smoking status and anthropomorphic measurements. Prevalent CVD was obtained by self-report or through general practitioners or hospital discharge reports.

Blood pressure was measured with a sphygmomanometer located on the right arm after a period of rest and in sitting position. Blood pressure was measured twice and the mean of two measurements was used. Hypertension was defined as systolic blood pressure ≥ 140 mmHg, or diastolic blood pressure ≥ 90 mmHg or use of anti-hypertensive medication. Blood pressure was measured twice in sitting position with a sphygmomanometer. Diabetes was defined as a fasting glucose level of ≥ 7.0 mmol/L or use of anti-diabetic medication. In women with POI we also used self-reported diabetes for the definition of diabetes. The National Cholesterol Education Program (NCEP) definition was used to determine MetS [31]. According to this definition MetS is present when ≥ 3 of the following features are present: waist circumference ≥ 88 cm, fasting glucose ≥ 6.1 mmol/L, BP > 130/85 mmHg, high density lipoprotein cholesterol (HDL-C) < 1.3 mmol/L, triglyceride (TG) ≥ 1.7 mmol/L. Fasting serum was obtained for glucose and lipid profile testing. Spare serum samples were stored at −80°C. Extensive information on the laboratory assessments are previously reported elsewhere [25,32,33].

A sub-group of POI patients, who also participated in the CREw-IMAGO study (Cardiovascular RiskprofilE—IMaging and gender-specific disOrders study), underwent PWV measurements [34],. These measurements were also performed in a sub-group of the controls who were screened during a fixed period of time in the Rotterdam Study [35]. The PWV was measured in supine position of the patients with the use of automatic devices; in women with POI the Arteriograph^™^ (TensioMed, Budapest, Hungary) was used [36] and in the controls the Complior Artech Medical was used [35,37].

Subclinical atherosclerosis is assessed by the bilateral measurement of cIMT, which can be used as a predictor for future cardiovascular events [38]. The definition of cIMT is the distance in micrometer between the lumen intima and the media-adventitia and measured three times at both sides over 1 centimeter length and at least 0.5 centimeters proximal of the bifurcation of the common carotid artery, or at the beginning of the dilatation of the distal common carotid artery across a length of 1 centimeter [25,39,40]. The mean of the mean of the right and left carotid arteries was used for the analysis. Ultrasound measurements were performed by one trained professional per research center. Several devices were used for ultrasounds in women with POI: the Panasonic CardioHealthStation and the ToshibaAplioArtida Medical System. In controls the ATL Ultra Mark IV transducer was used [40].

### Risk and health scores

In both groups we calculated the 10 year-CVD risk (FRS) for females and the CHS. The FRS was based on: age, systolic blood pressure, anti-hypertensive medication, HDL-C, total cholesterol, smoking and the presence of type II diabetes. Low risk was defined as a 10-year CVD risk < 10%, intermediate risk as CVD risk between 10–20% and > 20% was marked as high 10-year risk for CVD. In addition, we calculated the CHS in both study populations [26,27]. The CHS was introduced by the American Heart Association and incorporates health aspects (total cholesterol and glucose serum levels, blood pressure and BMI) and behavioral aspects (smoking, dietary intake and physical activity) [27]. Information on 2 out of the 7 factors (dietary intake and physical activity) was unfortunately not recorded similar or in detail in both study groups. It also has been noticed that data on dietary intake and physical activity is prone to sampling variability and misclassification [41,42]. Therefore, we calculated a composite CHS based on the 5 available parameters and assessed the mean CHS and performance of cases and controls on each of the health metric. All patients were divided into having a poor, intermediate or ideal health metric, according to the definitions proposed by the American Heart Association’s [27].

### Statistical analyses

A total of 148 women with POI underwent a cardiovascular screening in the current study. Our aim was to match each case with a control from the Rotterdam Study cohort, using propensity score matching (PSM) greedy approach with R software. PSM was based on a logistic regression model that includes POI versus no POI as a dichotomous outcome and age and BMI as covariate under study. Hosmer-Lemeshow test was used to evaluate goodness-or-fit of the models. Transformation of age and BMI was used based on lowess graph in order to choose the best fitting model. Standardized differences and plots of propensity scores distribution between POI and control group, before and after matching procedure, were made to evaluate the balance achieved. The PSM matchings procedure resulted in the successful matching of 123 women with POI and 123 controls.

Baseline characteristics were presented as means (with standard deviation) for data with a normal distribution or median (with interquartile ranges (IQR)) otherwise. Student t-tests and Mann-Whitney-U tests were performed for normally and non-normally distributed continuous data. Categorical variables were presented as number and percentage (%) of the total group of patients from which data was available. Chi-square tests or Fisher-Exact test were performed for categorical variables.

We used linear regression analyses to assess differences in cIMT after log transformation (log10) to obtain a normal distribution. We built several models to adjust for confounders: model 1 (no additional adjusting), model 2 (additional adjustments for smoking (never/ever), systolic blood pressure, education (low/intermediate/high) and medical center of origin (three university medical centers)), model 3 (adjustment for model 2 and HRT use (ever/never)) and model 4 (adjustments for model 3, lipid lowering medication (yes/no) and anti-hypertensive medication (yes/no). We also performed linear regression analyses to investigate whether reproductive age (menarche until last menstruation) or years between last menstruation and the current cardiovascular screening were associated with cIMT (used models: model 1 and a model with HRT use (yes/no) only). Results were expressed as regression coefficients (β) with corresponding 95% confidence intervals (95% CI) and p-values. We also performed a sensitivity analyses, aiming to detect any differences in POI patients with and without HRT use. A two-sided p-value of < 0.05 was considered significant. All analyses (with exclusion of the PSM procedure) were conducted using Statistical Package for Social Sciences (SPSS) version 25. (SPSS Inc. Chicago, IL, USA).

## Results and discussion

### Baseline characteristics

**Fig 1:**
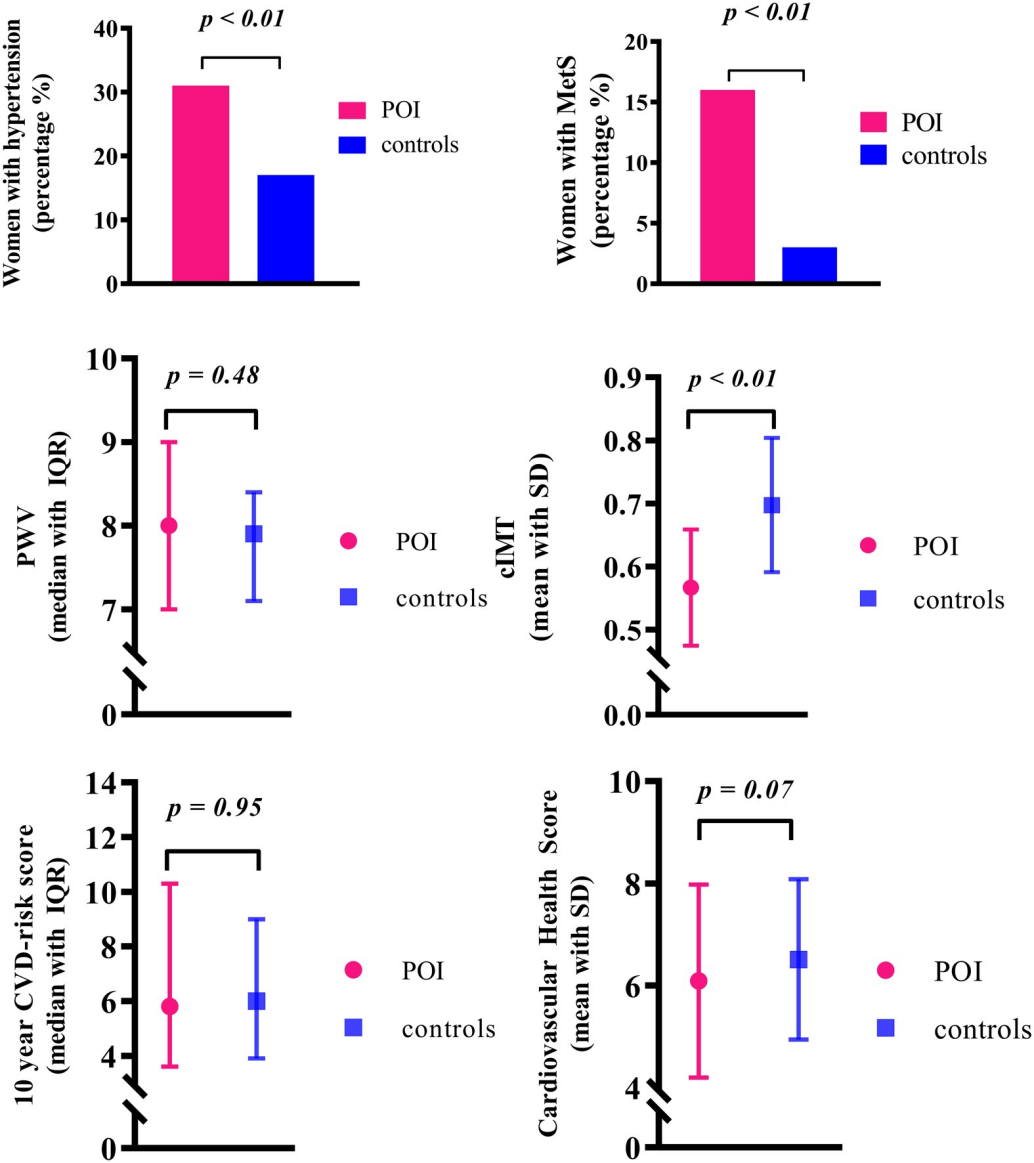
Cardiovascular parameters and outcomes for cardiovascular disease in middle age women with POI and age and BMI-matched controls. Abbreviations: cIMT = carotid intima media thickness, CVD = cardiovascular disease, IQR = inter-quartile range, MetS = metabolic syndrome, p = p-value, SD = standard deviation.

A total of 123 women with POI were matched for age and BMI with 123 female controls. Baseline characteristics of both women with POI and controls are depicted in Table 1. Women with POI presented with a higher prevalence of some of the cardiovascular risk factors, such as: hypertension, systolic blood pressure, diastolic blood pressure and MetS (Fig 1). On the other hand lipids, glucose, pulse wave velocity and prevalence of diabetes were similar in both groups (Table 1, Fig 1). Women with POI were more often treated with anti-hypertensive medication, but less often with lipid lowering medication compared to the controls. All characteristics of women who have and have not ever used HRT were compared, but did not differ significantly (S1 Table).

**Table 1:** Baseline characteristics of women with POI compared to age- and BMI-matched controls.

	POI (n = 123)	Control (n = 123)	P-value
**General parameters**			
Age (years)	49.0 (4.3)	49.4 (3.9)	0.43
Ethnicity (Caucasian)	105 (86%)	107 (88%)	0.70
Ever smoker	68 (57%)	79 (65%)	0.20
Age at menarche (years)	12.8 (1.5)	13.1 (1.6)	0.18
Postmenopausal status	123 (100%)	42 (34%)	**<0.01**
HRT use (yes)	77 (68%)	10 (8%)	**<0.01**
**Anthropometrics**			
BMI (kg/m^2^)	24.5 (21.7–27.8)	24.2 (22.1–26.8)	0.54
Waist (cm)	90.0 (83.0–98.0)	80.7 (75.1–86.8)	**<0.01**
Hip (cm)	102.0 (95.8–107.0)	102.3 (98.0–108.4)	0.17
Waist-to-hip ratio	0.90 (0.85–0.93)	0.79 (0.75–0.83)	**<0.01**
**Education**			
Primary	2 (2%)	7 (6%)	**<0.01**
Lower/intermediate or lower vocational	17 (14%)	36 (30%)	
Intermediate vocational or higher general	47 (39%)	42 (34%)	
Higher vocational or university	54 (45%)	37 (30%)	
**Cardiovascular parameters**			
Systolic BP (mmHg)	124 (112–135)	120.0 (109.0–131.0)	**0.04**
Diastolic BP (mmHg)	81 (76–89)	78 (71–86)	**<0.01**
Hypertension	45 (37%)	21 (17%)	**<0.01**
Pulse wave velocity (m/s)	8.1 (7.1–9.4)	7.9 (7.1–8.4)	0.21
Total cholesterol (mmol/L)	5.7 (4.9–6.2)	5.3 (4.9–6.1)	0.12
HDL cholesterol (mmol/L)	1.7 (1.4–1.9)	1.6 (1.3–2.0)	0.39
LDL cholesterol (mmol/L)	3.4 (2.9–4.1)	3.2 (2.7–3.8)	0.18
Triglycerides (mmol/L)	1.0 (0.8–1.4)	1.0 (0.8–1.3)	0.70
Glucose (mmol/L)	4.9 (0.9)	5.0 (0.5)	0.28
Diabetes	5 (4%)	4 (3%)	0.73
Anti-hypertensive medication	28 (24%)	0 (0%)	**<0.01**
Anti-hypercholesterolemia medication	2 (1.4%)	17 (14%)	**<0.01**
MetS (NCEP definition)	19 (16%)	4 (3%)	**<0.01**
History of CVD	2 (2%)	2 (2%)	1.00

Values are displayed as means (standard deviation) or medians (interquartile range), or as numbers (percentage). Differences were tested with Student’s T-test or Mann-Whitney-U for continuous variables, Chi-square or Fisher’s exact tests were used for categorical variables.

Abbreviations: BP = blood pressure, CVD = cardiovascular disease, HDL = high density lipoprotein, kg/m^2^ = kilograms per square meter, LDL = low density lipoprotein, m/s = meter per second, MetS = metabolic syndrome. mmHg = millimeters of mercury, mmol/L = millimole per liter, n = number of patients.

### CIMT measurement

CIMT, an early sign of atherosclerosis, was lower in women with POI (550 μm (500–615)) compared to controls 684 μm (618–737)) (β = -0.21 (95% CI: -0.25 − -0.17) with p < 0.01). This remained consistent in various multivariate models in which we corrected for additional confounders (Table 2). Amongst women with POI, CIMT also did not associate with the timespan between menarche and stopped menses (reproductive age) (β = -0.01 (95%CI: -0.01–0.01) with p = 0.93), nor the time between stopped menses and the current screening (β = 0.01 (95%CI: -0.01–0.01) with p = 0.44). This remained not significant after additional correction for HRT (β = -0.04 (95%CI: -0.11–0.04) with p = 0.33 and (β = -0.02 (95%CI: -0.09–0.06) with p = 0.65), respectively).

**Table 2:** Lower cIMT in women with POI compared to age- and BMI-matched controls: Adjusted regression analysis.

	β	95% Confidence Interval	p-value
**cIMT (logIMT)**			
**Model 1**	-0.21	-0.25–0.17	**<0.01**
**Model 2**	-0.16	-0.26–0.06	**<0.01**
**Model 3**	-0.16	-0.27–0.06	**<0.01**
**Model 4**	-0.20	-0.31–0.08	**<0.01**

**Model 1**: no additional adjustments

**Model 2**: additional adjustments for smoking (never/ever), systolic blood pressure, education (low/intermediate/high) and medical center of origin (three university medical centers).

**Model 3**: additional adjustments for Model 2 + HRT use (ever/never)

**Model 4**: additional adjustments for Model 3 + lipid lowering medication (yes/no), anti-hypertensive medication (yes/no).

Abbreviations: β = unstandardized regression coefficient, LogIMT is the natural logarithm of carotid IMT(cIMT); a transformation due to a non-normal distribution.

### Cardiovascular risk and cardiovascular health scores

**Fig 2:**
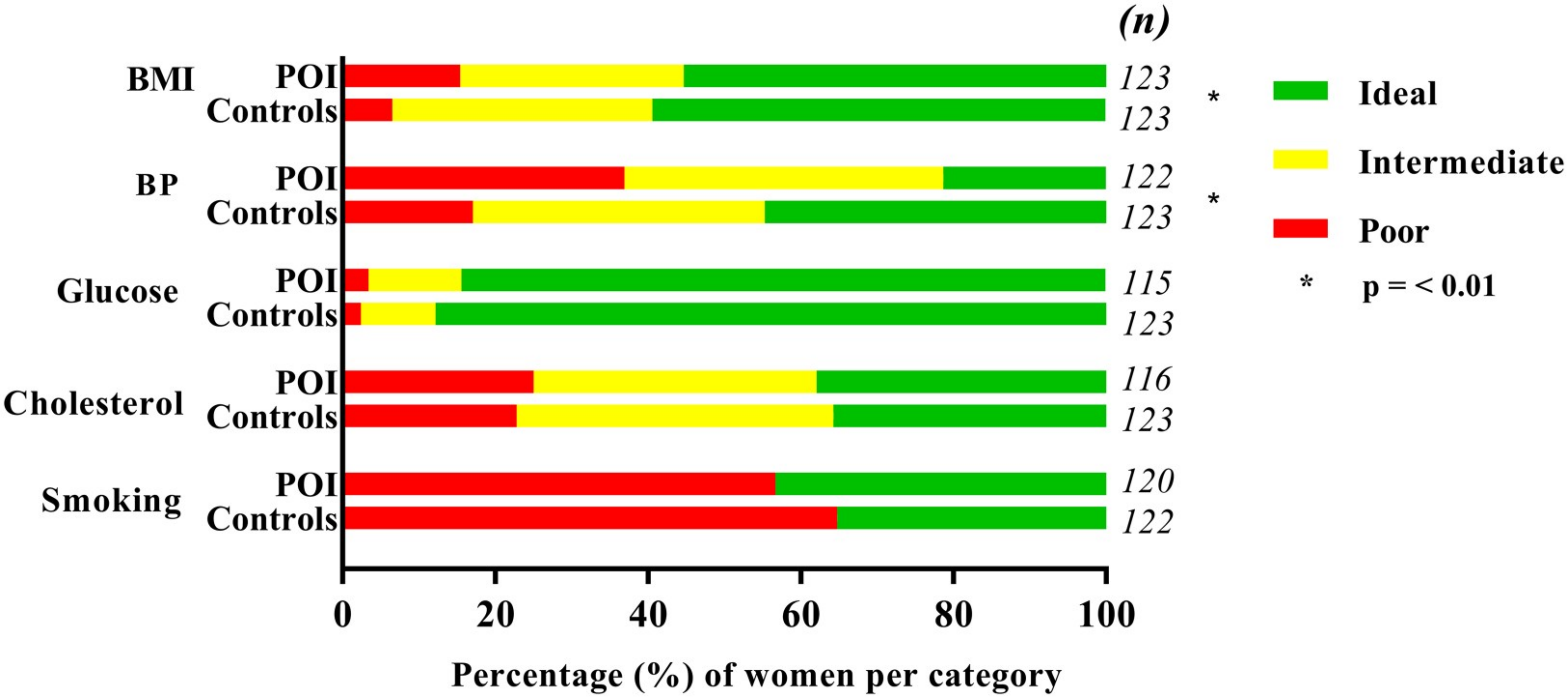
Risk groups of 10 year cardiovascular disease risk in women with POI and controls. Low risk: < 10%, intermediate risk 10–20%, high risk > 20%. Above the columns the numbers of patients which each column represent are listed. Abbreviations: p = p-value, POI: premature ovarian insufficiency.

**Fig 3:**
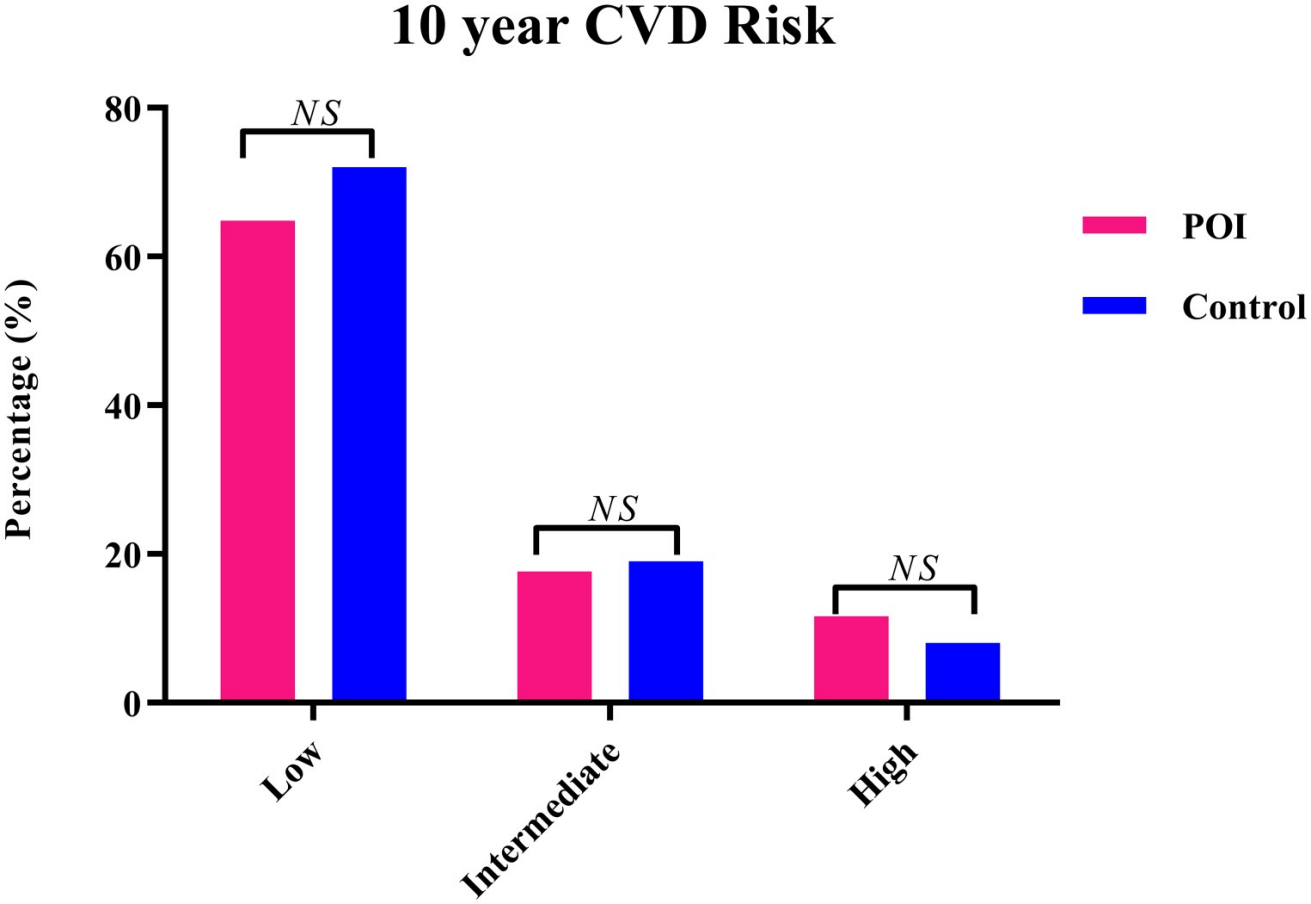
Graph of the available components of the cardiovascular health metrics score in women with POI versus healthy controls. Prevalence (%) of poor, intermediate and ideal cardiovascular heath metrics in women diagnosed with POI and controls. Abbreviations: BMI: body mass index, BP: blood pressure, (n) = number of patients included in each group per individual metric, p = p-value, POI: premature ovarian insufficiency.

The distribution of low, intermediate and high 10 years risk according to the Framingham risk score was similar in women with POI and controls (Fig 1). When comparing the continuous 10-year CVD risk score, we observed similar risk scores between the two study populations: median FRS in women with POI: 5.9% (IQR: 3.7–10.6) and in controls: 6.0% (IQR: 3.9–9.0), with p-value = p = 0.31. The mean CHS was 6.1 (1.9) in women with POI and 6.5 (1.6) in controls, with p-value = 0.07 (Fig 1). Within the three risk score groups of the FRS we did not detect any significant difference between women with POI and controls (Fig 2). The outcomes of individual components of the CHS are divided into ideal, intermediate or poor values and presented in Fig 3. BMI and blood pressure groups only differed significantly between the two groups. Controls were more often assigned to healthier categories (Fig 3). Cardiovascular risk and cardiovascular health scores of women with and without HRT use were compared, but did not differ significantly (S1 Table).

In the current cross-sectional study, we compared cardiovascular profiles in a cohort of well phenotyped women at middle age and previously diagnosed with POI, with age- and BMI-matched population based controls. POI was diagnosed before 40 years of age and confirmed with a standardized endocrine screening 8 years (IQR: 6–9) before the current screening. Confirmation of POI diagnose was achieved at an average age of 39 years (IQR: 37–43). We assessed cardiovascular risk factors, cIMT (as a surrogate marker for atherosclerosis) and cardiovascular risk and health scores. An increased prevalence of hypertension and MetS was observed in women with POI compared to controls, but no increased risk of other cardiovascular risk factors (including lipid profile, diabetes), and a lower cIMT which remained significant after additional correction for potential confounders. Ten-year CVD risk and overall performance concerning cardiovascular health metrics (CHS) were found to be similar in women with POI compared to the general population.

In previous epidemiological studies it has been convincingly demonstrated that age of menopause is associated with CVD risk and mortality [5]. In accordance with these findings, we confirm in the current study the presence of several risk factors in women with the most extreme form of early menopause, i.e. POI. The observed increased waist circumference, along with the increased incidence of hypertension and MetS in women with POI would suggest a predisposition to more abnormal surrogate markers for cardiovascular disease. This, however, is not the case in the current analysis since we observe neither an increased preclinical signs of atherosclerosis, nor an increased 10-year CVD risk.

Given these study results the question arises: How certain are we concerning a possible association between spontaneous POI and increased CVD? Several hypotheses have been proposed to explain the possible association between the aforementioned. Firstly, the significantly extended reduced estrogen exposure may result in loss of its cardioprotective properties resulting in an increase in CVD [43]. Blood pressure increases with age due to the increased sympathetic nervous system activity, which is observed in aging humans [44]. Menopause related blood pressure increase could as well be influenced by changing estrogen/androgen ratios, resulting in a relatively hypo-estrogenic and hyper-androgenic state [45]. Estrogen deprivation was also associated with increased pro-inflammatory markers such as interleukins, which are associated with diabetes, atherosclerosis and cardiovascular disease [46]. Secondly, genetically determined accelerated somatic aging may manifest by both early deterioration of cardiovascular health along with premature ovarian aging [47–49]. Another hypothesis states that ovarian aging may be the result of the worsening of cardiovascular health, which could translate into diminished ovarian vascularization due to atherosclerotic lesions [50]. Regardless of the uncertainty surrounding these hypotheses, the association between early menopause and cardiovascular disease was most convincingly confirmed in women with surgically induced POI which may be interpreted as estrogen deprivation being the predominant determinant of increase cardiovascular risk [51].

CVD risk factors hypertension and MetS [52,53] are increasingly present in post-menopausal women and contribute to the negative association between menopause status and CV health. We were able to confirm such associations in current study population of women with POI. Before menopause, blood pressure is lower in women compared to men of similar age [54] but after menopause women undergo an age-related blood pressure increase [55,56].

The PWV is a surrogate marker for arterial stiffness which independently predicts a first CV event and even mortality [57,58]. An earlier study on PWV in women with POI compared to controls also revealed a similar arterial stiffness amongst women with POI and controls. These women, however, were younger (approximately ten years) compared to the women in our study group, and were therefore exposed to a decreased estrogen status for a shorter period of time [59]. In the current study PWV was similar in both groups, which implies no signs of increased arterial stiffness in women with POI.

Furthermore, we measured cIMT with ultrasound during the cardiovascular screening as a proxy for preclinical atherosclerosis. We observed that cIMT was lower in women with POI compared to controls [25]. Adjustments for potential confounders did not alter the observed lower cIMT. CIMT measurements were performed with various machines in women with POI, but yielded similar results across the different research centers (data not shown). However, despite the consistency amongst women with POI across centers (data not shown), caution is warranted in interpreting the cIMT results. Supporting the absence of an increased cIMT in women with POI, the Framingham 10-year CVD risk and the CHS score yielded no differences between POI and age- and BMI-matched controls to the detriment of the health of middle-aged women with POI, despite the presence of increased CVD risk factors.

One of the potential confounders used in the analyses of cIMT was HRT use. HRT is prescribed until the age of natural menopause in women with POI in order to treat symptoms of low estrogen concentrations (e.g. vasomotor symptoms) [16]. This recommendation, however, is often not followed [60]. Women often prematurely stop HRT or start treatment many years after initial diagnosis. To evaluate any differences in POI patients with and without the ever use of HRT we compared their baseline characteristics, intermediate cardiovascular outcomes and CV scores. Results of these analyses yielded no significant differences. Data on the exact dose, formulation, period of use were limited; therefore we chose to use data on HRT as a dichotomous variable only. Hence, we were unable to make a robust distinction between brief use of HRT or HRT use until the age of natural menopause. In a previous study performed by our group using a different cohort of POI patients, we did observe a correlation between prolonged estrogen deprivation and a Framingham 30-year risk of CVD risk at the time of their POI diagnosis [43]. These women were, however, 14 years younger compared to women with POI participating in the current study [43].

A multitude of risk scores are available for cardiovascular endpoints. We specifically aimed to use a 10-year risk prediction score to avoid an underestimation of the cardiovascular disease risk. We avoided using risk scores which do not take unfatal myocardial infarction and/or stroke into account, such as SCORE, Reynolds risk score and ATPIII [61–63]. For the use of the accurate use of the QRISK2/3 score we would need more information on kidney disease, rheumatoid arthritis and atrial fibrillation [64,65]. In the ASCVD risk score (ACC/AHA), which also predicts a 10-year risk, stroke is also taken into account as one of the endpoints [66]. However, a recent meta-analysis did not report an increased fatal and non-fatal stroke risk in women with premature ovarian insufficiency [23]. Additional to the fore mentioned we preferred a risk score in which angina pectoris was also taken into account, since we focus on young women who in the general population have a low risk for manifest CVD. Hence the choice for the 10-year Framingham risk score.

Various hypotheses may be proposed to explain the observed results of the current study, which seem to contradict the increased cardiovascular disease and mortality previous studies reported [5,8,10]. The investigated women with a POI may still be too young (mean age 49 years) to develop arterial stiffness or an increased cIMT and very likely to develop CVD endpoints [67–69]. A Dutch study reports the cumulative incidence of CVD in Dutch women, which is still low at 55 years old, even lower compared to men [69]. It therefore may be required to extend the follow-up time to detect preclinical signs of atherosclerosis or to be certain on the absence of increased CVD risk regardless of increased cardiovascular risk factors in our study population of women with POI. Various potential women-specific confounders may also explain the lack of observed distinct abnormalities in various cardiovascular parameters, such as lifestyle and psycho-social factors. The current study population of women with POI might have improved their lifestyle after receiving their diagnosis and counseling concerning potential future health risks. This could counterbalance detrimental effects of the risk factors observed, such as hypertension and MetS. Another option would be that the protective effects of HRT influence the health of our patient group. It could also be the case that we are overseeing certain women specific surrogate markers which differ between the POI population and controls, since distinct differences have been observed between risk factors and clinical manifestation of CVD comparing males and females [61, 70].

One of the strengths of this study is that well phenotyped women with POI were prospectively included in the current cohort, follow up study. These women included, underwent a comprehensive standardized screening to confirm their diagnosis. In addition, we were able to include a relatively large number of women with this low prevalent condition. The current study is an extension of our previous work in women with POI [22,25]. Firstly, we continued our comprehensive cardiovascular screening of well-phenotype women diagnosed with POI and added over 60 patients to our POI cohort. Secondly, we expanded the cardiovascular screening by also assessing endothelial dysfunction as tested by PWV measurements. Thirdly, for the current study we selected a control group using an individual matching procedure regarding age and BMI regardless of their menopausal status, which minimalizes the influence of the classical risk factor BMI on CVD. And fourthly we evaluated the 10-year CVD risk (FRS) and a limited version of the CHS [26].

Several limitations of the current study should also be mentioned. We were unable to match all patients with controls due to an age differences between our patient (≥ 40 years) and the control group (≥ 45 years). The PWV was measured with two different devices (however, both devices were automated) and different researchers. Limited statistical power prevents us from definitive conclusions on FRS and CHS in women with POI compared to controls. It should be noted that the size of the population could still be insufficient to detect subtle differences, if present at all. A post hoc power calculation performed on the endpoints of this study estimated the percentage of certainty with which we were able to detect differences. This resulted in a power of 100% for cIMT and only 47% of CHS and 52% for FRS (https://clincalc.com/stats/Power.aspx). Because of lack of power and lack of detailed data regarding history of HRT use, great caution is needed in interpreting the CHS and FRS data.

Future long term, follow up studies monitoring aging of women previously diagnosed with POI will provide the final answer to the question whether these women, are truly at increased risk for future CVD. In addition, more robust data regarding the possible cardioprotective effect of long term HRT use is urgently needed. Based on the current data were not able to provide evidence concerning premature atherosclerosis or increased risk for future CVD in middle-aged women with POI. Future studies must provide insight in how their CV profile changes over time and investigate various cardiovascular endpoints over time such as ischemic heart disease, stroke, CVD mortality and all-cause mortality.

## Conclusion

Despite increased cardiovascular risk factors present in middle aged women with premature ovarian insufficiency, we did not observe worse: cIMT, 10 year CVD risk according to the Framingham risk score, nore worse cardiovascular health metrics (CHS), compared to age- and BMI-matched female controls from the general population. Continued follow-up screening is warranted to investigate ‘hard’ cardiovascular outcomes.

## Supporting information

S1 Appendix (DOCX)

S1 Table Baseline characteristics—Women with POI: Women who have ever used HRT versus women who have never used HRT. (DOCX)
