# Supplementary material for: The cardiovascular risk profile of middle age women previously diagnosed with premature ovarian insufficiency: A case-control study
Source: PLoS One. 2020 Mar 5;15(3):e0229576. doi: 10.1371/journal.pone.0229576 (PMC7058320; doi:10.1371/journal.pone.0229576)
Supplement: S1 Appendix — (DOCX) [file pone.0229576.s001.docx]

## S2 Appendix. Membership of the CREW consortium.

The CREW consortium consists of (in alphabetical order):

Yolande Appelman^1^, Sara Baart^2,3^, Laura Benschop^2,3^, Eric Boersma^2^, Laura Brouwers^3,4^, Ricardo Budde^2^, Suzanne Cannegieter^5^, Nadine Daan^4^, Veerle Dam^3,6^, Rene Eijkemans^4,6^, Bart Fauser^4^, Michel Ferrari^5^, Arie Franx^2,4^, Christianne de Groot^1^, Marlise Gunning^3,4^, Annemieke Hoek^7^, Erik Koffijberg^6,8^, Wendy Koster^2^, Mark Kruit^5^, Giske Lagerweij^3,6^, Nils Lambalk^1^, Joop Laven^2^, Katie Linstra^2,3,5^, Aad van der Lugt^2^, Angela Maas^9^, Antoinette Maassen van den Brink^2^, Cindy Meun^2,3^, Saskia Middeldorp^10^, Karel G.M. Moons^6^, Bas van Rijn ^2,4^, Jeanine Roeters van Lennep^2^, Jolien Roos-Hesselink^2^, Luuk Scheres^3,10^, Yvonne T. van der Schouw^6^, Eric Steegers^2^, Regine Steegers^2^, Gisela Terwindt^5^, Birgitta Velthuis^3,4^, Marieke Wermer^5^, Bart Zick^2,5^, Gerbrand Zoet^3,4^.

^1^ Amsterdam UMC – location VUmc, Amsterdam, the Netherlands

^2^ Erasmus University Medical Center, Rotterdam, the Netherlands

^3^ Netherlands Heart Institute, Utrecht, the Netherlands

^4^ University Medical Center Utrecht, Utrecht, the Netherlands

^5^ Leiden University Medical Center, Leiden, the Netherlands

^6^ Julius Center, Utrecht, University Medical Center, Utrecht, the Netherlands

^7^ University Medical Center Groningen, Groningen, the Netherlands

^8^ University of Twente, Enschede, the Netherlands

^9^ Radboud University Medical Center, Nijmegen, the Netherlands

^10^ Amsterdam UMC – location AMCenter, Amsterdam, the Netherlands
