# Supplementary material for: The cardiovascular risk profile of middle age women previously diagnosed with premature ovarian insufficiency: A case-control study
Source: PLoS One. 2020 Mar 5;15(3):e0229576. doi: 10.1371/journal.pone.0229576 (PMC7058320; doi:10.1371/journal.pone.0229576)
Supplement: S1 Table — (DOCX) [file pone.0229576.s002.docx]

## S1 Table. Baseline characteristics of middle age women previously diagnosed with POI: separate for women who have ever or never used HRT.

|  | | **POI HRT+ (n = 77)** | **POI HRT- (n = 37)** | **p-value** |
| --- | --- | --- | --- | --- |
| **General parameters** | |  |  |  |
| Age (years) | | 48.7 (3.6) | 50.1 (5.7) | 0.12 |
| Ethnicity (Caucasian) | | 66 (86%) | 33 (92%) | 0.37 |
| Ever smoker |  | 45 59%) | 22 (61%) | 0.85 |
| Age at menarche (years) | | 12.7 (1.5) | 12.8 (1.3) | 0.67 |
| Age of amenorrhea | | 34.4 (5.4) | 34.5 (5.3) | 0.93 |
| Time between menarche and last menses | | 21.2 (5.7) | 22.4 (5.5) | 0.34 |
| Time between last menses and current screening | | 14.4 (6.9) | 15.9 (8.3) | 0.37 |
| **Anthropometrics** | |  |  |  |
| BMI (kg/m^2^) | | 24.8 (22.8-27.9) | 23.8 (21.1-27.7) | 0.15 |
| Waist (cm) | | 91.5 (83.0-97.8) | 88.0 (79.0-100.0) | 0.69 |
| Hip (cm) | | 102.0 (97.0-107.0) | 100.0 (91.0-107.0) | 0.42 |
| Waist-to-hip ratio | | 0.90 (0.84-0.93) | 0.89 (0.86-0.93) | 0.81 |
| **Education** | |  |  |  |
| Primary | | 1 (1%) | 1 (3%) | 0.88 |
| Lower/intermediate or lower vocational | | 11 (15%) | 5 (14%) |  |
| Intermediate vocational or higher general | | 32 (42%) | 13 (36%) |  |
| Higher vocational or university | | 32 (42%)) | 17 (47%) |  |
| **Cardiovascular parameters** | |  |  |  |
| Systolic BP (mmHg) | | 122 (111-134) | 125 (117-140) | 0.19 |
| Diastolic BP (mmHg) | | 80 (76-98) | 85 (75-91) | 0.23 |
| Hypertension | | 24 (31%) | 17 (46%) | 0.12 |
| Pulse wave velocity (m/s) | | 7.8 (7.1-9.4) | 8.1 (7.6-9.5) | 0.43 |
| Total cholesterol (mmol/L) | | 5.7 (5.0-6.3) | 5.4 (4.8-6.3) | 0.53 |
| HDL cholesterol (mmol/L) | | 1.7 (1.3-2.0) | 1.7 (1.4-1.8) | 0.87 |
| LDL cholesterol (mmol/L) | | 3.5 (3.0-4.2) | 3.4 (2.6-4.3) | 0.89 |
| Triglycerides (mmol/L) | | 1.1 (0.8-1.5) | 1.0 (0.8-1.2) | 0.27 |
| Glucose (mmol/L) | | 4.9 (0.9) | 4.8 (0.7) | 0.37 |
| Diabetes | | 3 (4%) | 2 (5%) | 0.58 |
| Anti-hypertensive medication | | 13 (18%) | 12 (33%) | 0.06 |
| Anti-hypercholesterolemia medication | | 0 (0%) | 2 (5%) | 0.10 |
| MetS (NCEP definition) | | 13 (17%) | 4 (11%) | 0.39 |
| History of CVD | | 0 (0%) | 2 (5%) | 0.10 |
| **Outcomes** | |  |  |  |
| Mean cIMT | | 550.0 (500.0-600.0) | 550.0 (500.0-650.8) | 0.74 |
| FRS | | 6.0 (3.6-10.2) | 6.4 (4.4-11.2) | 0.39 |
| CHS | | 6.0 (1.8) | 5.0 (2.1) | 0.61 |

S1 Table Legend: A total of 9 women with POI did not recall their HRT use status; their baseline characteristics are excluded from this table. Values are displayed as means (standard deviation) or medians (interquartile range), or as numbers (percentage). Differences were tested with Student’s T-test or Mann-Whitney-U for continuous variables, Chi-square or Fisher’s exact tests were used for categorical variables. Abbreviations: + = ever use presence, - = ever use absent BP = blood pressure, cIMT = carotid intima media thickness, CHS = cardiovascular health score, cm = centimeter, CVD = cardiovascular disease, FRS = Framingham risk score (in %), HDL = high density lipoprotein, kg/m^2^ = kilograms per square meter, LDL = low density lipoprotein, MetS = metabolic syndrome, m/s = meter per second, mmHg = millimeters of mercury, mmol/L = millimole per liter, n = number of patients.
